# Different Survival of Barcelona Clinic Liver Cancer Stage C Hepatocellular Carcinoma Patients by the Extent of Portal Vein Invasion and the Type of Extrahepatic Spread

**DOI:** 10.1371/journal.pone.0124434

**Published:** 2015-04-29

**Authors:** Dong Hyun Sinn, Ju-Yeon Cho, Geum-Youn Gwak, Yong-Han Paik, Moon Seok Choi, Joon Hyeok Lee, Kwang Cheol Koh, Seung Woon Paik, Byung Chul Yoo

**Affiliations:** Department of Medicine, Samsung Medical Center, Sungkyunkwan University School of Medicine, Seoul, South Korea; Kaohsiung Chang Gung Memorial Hospital, TAIWAN

## Abstract

Portal vein invasion (PVI) and extrahepatic spread (ES) are two tumor-related factors that define advanced stage in the Barcelona Clinic Liver Cancer (BCLC) staging system (BCLC stage C), and the recommended first line therapy in this stage is sorafenib. However, the extent of PVI and the type of ES may affect patient prognosis as well as treatment outcome. This study analyzed survival of BCLC stage C HCC patients in order to see whether sub-classification of BCLC stage C is necessary. A total of 582 treatment naïve, BCLC stage C HCC patients [age: 54.3 ± 10.8 years, males = 494 (84.9%), hepatitis B virus (458, 78.7%)], defined by PVI and/or ES, were analyzed. Extent of PVI was divided into none, type I-segmental/sectoral branches, type II-left and/or right portal vein, and type III-main portal vein trunk. Type of ES was divided into nodal and distant metastasis. The extent of PVI and type of ES were independent factors for survival. When patients were sub-classified according to the extent of PVI and type of ES, the median survival was significantly different [11.7 months, 5.7 months, 4.9 months and 2.3 months for C1 (PVI-O/I without distant ES), C2 (PVI-II/III without distant ES), C3 (PVI-0/I with distant ES), and C4 (PVI-II/III with distant ES), respectively, *P* = 0.01]. Patients’ survival was different according to the treatment modality in each sub-stage. Sub-classification of BCLC stage C according to the extent of PVI and type of ES resulted in a better prediction of survival. Also, different outcome was observed by treatment modalities in each sub-stage. Sub-classification of BCLC stage C is required to minimize heterogeneity within the same tumor stage, that will help better predict survival and to select optimal treatment strategies.

## Introduction

Hepatocellular carcinoma (HCC) is one of the most common causes of cancer-related mortality worldwide [1]. Many factors are associated with survival of patients with HCC, and selection of optimal treatment strategy can improve patient survival [2]. The choice of treatment modality should consider the size and number of the tumor, tumor location, anatomical considerations, and liver functions [2]. Tumor staging is intended to help estimate patients prognosis and guide decision making for treatment [3]. There are several staging system proposed for HCC, but the Barcelona Clinic Liver Cancer (BCLC) staging system is unique among the other staging systems used for HCC that each stage simultaneously links treatment strategy [3].

The advanced stage, BCLC stage C, is defined for the group of patients with adverse predictors, which includes patients with cancer-related symptoms, portal vein invasion (PVI), extrahepatic spread (ES), or a combination of these factors, in patients with preserved liver function, defined by Child-Pugh Class A or B [3]. Of those, PVI and ES are two tumor-related factors that define advanced stage. However, PVI can involve only segmental or sectoral branch, or may extend to left or right main portal vein, or to main trunk and beyond. These different types of PVI cannot be considered to have same disease course [4]. Also, ES can be divided into nodal and distant metastasis, and the American Joint Committee on Cancer (AJCC)/International Union Against Cancer (UICC) tumor-node-metastasis (TNM) staging system separately assesses nodal and distant metastasis [5].

Sorafenib is the only proven, current standard treatment for BCLC stage C [3]. However, a wide range of treatment modalities, including resection [6, 7], transarterial chemoembolization (TACE) [8], radiation, hepatic arterial infusion chemotherapy [9], radioembolization [10], systemic cytotoxic chemotherapy [11], TACE plus radiation [12], TACE plus sorafenib [13], sorafenib plus radiation [14], and liver transplantation after the combined use of locoregional therapy [15], are still being employed in the real world on an empirical basis. Furthermore, the reported median survival of patients with advanced HCC after resection (27.8 months) [6], drug-eluting bead TACE (13.5 months) [8], or radioembolization (13.0 months) [16], was longer than the median survival of patients with advanced HCC after sorafenib therapy in Sorafenib HCC Assessment Randomized Protocol Trial (SHARP) (10.7 months) [17], and in the Asia-Pacific Study (6.5 months) [18]. In our previous study, patients with locally advanced HCC without distant metastasis who were treated with TACE plus radiation showed longer overall survival than patients who were treated with sorafenib [12]. In the study by Choi et al., TACE plus sorafenib was superior to sorafenib alone in patients with advanced HCC [13]. These findings indicate that advanced stage (BCLC stage C) includes a heterogeneous population that optimal treatment modality may not be sorafenib monotherapy for some patients.

Under these circumstances, sub-classification of the BCLC stage C may be necessary in order to better predict the patient outcome as well as to better guide optimal treatment modality. Therefore, this study was performed to analyze the survival of BCLC stage C HCC patients, sub-classify them according to tumor characteristics that determine survival, and finally compare the efficacy of different treatment options in each sub-stage.

## Methods

### Data source

The HCC registry of Samsung Medical Center, Seoul, South Korea, between January 2007 and December 2011 was reviewed. The HCC registry of Samsung Medical Center is a prospective registry that records clinical characteristics, tumor characteristics, as well as treatment information of every HCC patient diagnosed at Samsung Medical Center since January 2005. HCC was diagnosed by either histological or clinical evaluation based on the typical imaging findings and elevated serum alpha-fetoprotein levels [19]. After the patient was diagnosed with HCC, well-trained abstractors collected the patient’s data including age at diagnosis, gender, date of diagnosis, etiology, liver function (e.g., Child-Pugh class), tumor characteristics (e.g., number of tumors, maximal tumor size, presence and extent of PVI, and type of ES), tumor stage (both AJCC/UICC and BCLC stage), and initial treatment modality, in a prospective manner. During the study period, a total of 582 patients were classified into BCLC stage C because of PVI or ES. The study was reviewed and approved by the Institutional Review Board of Samsung Medical Center (IRB No: 2013-12-030). Because the study is based on the retrospective analysis of existing administrative and clinical data, the requirement of obtaining informed patient consent was waived by the Institutional Review Board of Samsung Medical Center. Patient records/information was anonymized and de-identified prior to analysis.

### Classification of PVI and ES, treatment and patient survival

For this study, the extent of PVI was classified as none, type-I (segmental or sectoral PVI), type-II (left and/or right main PVI), and type-III (main trunk invasion or beyond), based on radiological exam. Type of ES was classified as none, nodal, and distant metastasis. Nodal and distant metastasis was defined according to the AJCC 7^th^ editions [5]. Radiologically, nodal metastasis was defined positive if the lymph node short axis is ≥ 20 mm, according to the modified Response Evaluation Criteria in Solid Tumors (mRECIST) criteria [20].

Initial treatment modality was chosen by respective doctor. For this study, treatment was grouped into best supportive care, sorafenib and other modalities. Sorafenib was started using 400 mg of sorafenib twice daily, with dose modification according to toxicity as needed. Other modalities were composed of resection, transplantation, chemoembolization, systemic chemotherapy, radiation and experimental therapy (e.g. Brivanib). Surgical procedures were performed by experienced surgeons using standard surgical techniques for hepatectomy or transplantation. Chemoembolization was done using mixture of doxorubicin hydrochloride (Adriamycin; Dong-A Pharm, Seoul, Korea) and iodized oil (Lipiodol; Guerbet, Aulnay-sous-Bois, France). The doses of adriamycin and lipiodol were dependent on the tumor size and vascularity, with a maximum of 70 mg of adriamycin and 25 ml of lipiodol per session. Additional particulate embolization with 1- to 2-mm-diameter gelatin sponge pledgets (Cutanplast; MasciaBrunelli, Milan, Italy) was done when insufficient blockage of the tumor feeding arteries was evident.

Patient survival data was collected from the National Statistics Service, hence, all death at the time of survival assessment could be certified. All of the patients were followed up till death or July 31, 2013.

### Statistical analysis

Statistical analysis was performed using the chi-square test to compare discrete variables and the t-test for continuous variables. The survival rate was calculated and plotted using the Kaplan-Meier method. Differences in the incidence rate between the groups were analyzed using a log-rank test. To identify the factors associated with survival, multivariable analysis was performed using the Cox proportional hazard model for variables with *P* values of <0.05 on the univariable analysis. A *P* value of less than 0.05 was considered to be significant.

## Results

### Clinical characteristics and survival

The baseline characteristics of patients are shown in Table 1. The reason for BCLC stage C was the presence of PVI in 354 patients, the presence of ES in 106 patients, and a combination of these two factors in 122 patients. The median survival of patients in BCLC stage C was 6.4 months, and the range of survival was very wide (0 to 79 months). The survival rate was 51%, 33% and 13% at 6 months, 1 year and 3 years, respectively.

**Table 1:** Baseline characteristics.

Characteristics	N = 582
Male (n,%)	494 (84.9%)
Age (years, mean ± S.D)	54.3 ± 10.8
Diagnostic tools	
Imaging diagnosis	508 (87.3%)
Pathologic diagnosis	74 (12.7%)
Etiology	
Hepatitis B	458 (78.7%)
Hepatitis C	33 (5.7%)
Hepatitis B and C	10 (1.7%)
Others	81 (13.9%)
Child-Pugh class	
A	455 (78.2%)
B	127 (21.8%)
Tumor number	
Single	297 (51.0%)
Multiple	285 (49.0%)
Maximal tumor size	
< 10 cm	309 (53.1%)
≥ 10 cm	273 (46.9%)
Alphafetoprotein levels (ng/ml)^*^	1,379 (56–19,903)
Extent of portal vein invasion	
None	106 (18.2%)
I (segmental/sectoral)	260 (44.7%)
II (left and/or right main)	66 (11.3%)
III (main trunk or beyond)	150 (25.8%)
Extrahepatic spread	
None	354 (60.8%)
Node metastasis	68 (11.7%)
Distant metastasis^†^	160 (27.5%)

^*^Initial alphafetoprotein level was missing in 38 patients.

^†^Of the 160 patients with distant metastasis, 33 patients (20.6%) had concomitant nodal metastasis.

### Prognosis based on the extent of PVI

**Fig 1:**
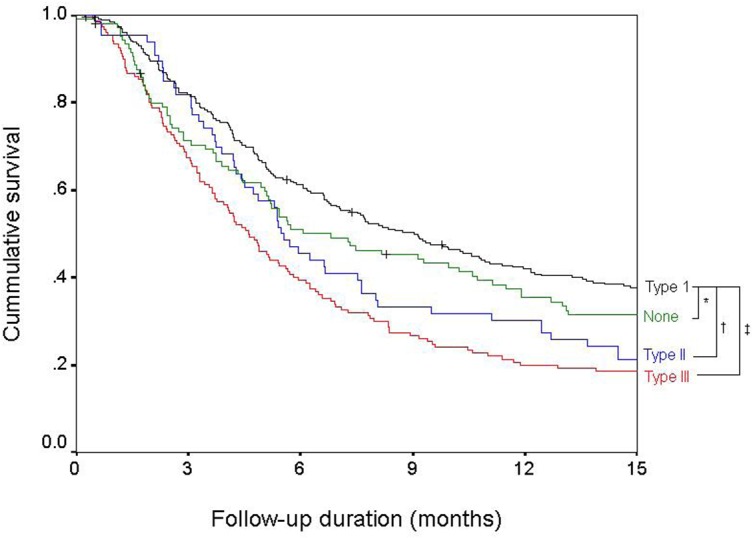
Survival of patients based on the extent of portal vein invasion. There was a significant survival difference based on the extent of portal vein invasion. The extent of PVI was classified as none (green), type-I (segmental or sectoral PVI, black), type-II (left and/or right main PVI, blue), and type-III (main trunk invasion or beyond, red). ^*^ *P* = 0.19; ^†^ *P* = 0.01; ^‡^ *P* < 0.01.

There was a significant survival difference based on the extent of PVI (Fig 1). Patients with type-I PVI had the best median survival of 9 months. The median survival of patients with type-II PVI [5.5 months; unadjusted hazard ratio (HR) (95% confidence interval (CI)): 1.47 (1.10–1.96), *P* = 0.01] and type-III PVI [4.6 months; unadjusted HR (95% CI): 1.73 (1.40–2.15), *P* < 0.01] was significantly worse than that of patients with type-I PVI. The survival of patients without PVI, but with ES [6.8 months; unadjusted HR (95% CI): 1.17 (0.91–1.51), *P* = 0.19] was not significantly different from the survival of patients with type-I PVI.

### Prognosis based on the type of ES

**Fig 2:**
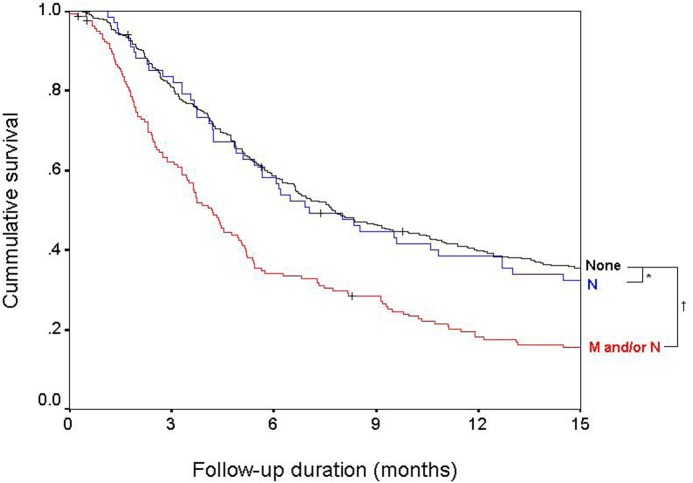
Survival of patients based on the type of extrahepatic spread. There was a significant survival difference based on the type of extrahepatic spread. Type of ES was classified as none (black), nodal metastasis only (N, blue) and distant metastasis with or without nodal metastasis (M and/or N, red). ^*^ *P* = 0.84; ^†^ *P* < 0.01.

Survival differed according to the type of ES (Fig 2). The median survival of patients with PVI but without ES was 7.7 months, which was not different from the median survival of patients with nodal metastasis [7.0 months; unadjusted HR (95% CI): 1.02 (0.77–1.36), *P* = 0.84]. However, survival was significantly worse for patients with distant metastasis [median survival: 4.2 months; unadjusted HR (95% CI): 1.74 (1.42–2.13), *P* < 0.01].

### Prognosis based on the extent of PVI and the type of ES

Child-Pugh Class, tumor size, extent of PVI and type of ES, AFP level were independent factors associated with survival (Table 2). Based on these two independent tumor related factors that define BCLC stage C, we sub-classified patients into sub-stages C1~C4 (Table 3). Sub-stage C1 includes the patients with PVI limited to the segmental or sectoral branch and ES limited to the nodal area. Sub-stage C2 includes the patients with PVI that extends beyond the right and/or left main branch without distant metastasis, and sub-stage C3 includes the patients with distant metastasis but PVI limited to the segmental or sectoral branch. Sub-stage C4 includes the patients with PVI that extends beyond the right and/or left main branch with distant metastasis. Based on the liver function (Child-Pugh class), each sub-stage was further classified into Cxa or Cxb, which indicates the Cx stage with Child-Pugh class A or Child-Pugh class B, respectively.

**Fig 3:**
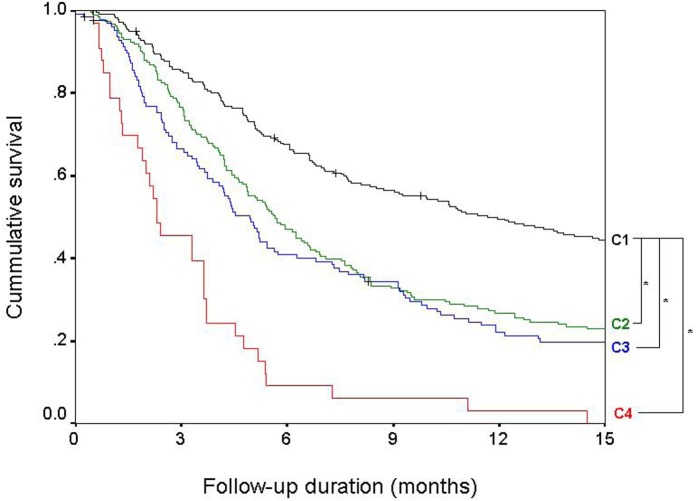
Survival of patients based on the sub-classification. Patient survival was significantly different based on sub-classification of BCLC stage C patients. Sub-stage C1 includes the patients with portal vein invasion limited to the segmental or sectoral branch and extrahepatic spread limited to the nodal area (black). Sub-stage C2 includes the patients with portal vein invasion that extends beyond the right and/or left main branch without distant metastasis (green), and sub-stage C3 includes the patients with distant metastasis, but portal vein invasion limited to the segmental or sectoral branch (blue). Sub-stage C4 includes the patients with portal vein invasion that extends beyond the right and/or left main branch with distant metastasis (red). ^*^ *P* < 0.01.

Patient survival was significantly different according to sub-stages within the BCLC stage C (Fig 3). The median survival of patients in sub-stage C1 (11.7 months) was significantly longer than the median survival of patients in sub-stage C2 [5.7 months; unadjusted HR (95% CI): 1.73 (1.40–2.13), *P* < 0.01], sub-stage C3 [4.9 months; unadjusted HR (95% CI): 1.89 (1.49–2.39), *P* < 0.01], and sub-stage C4 [2.3 months; unadjusted HR (95% CI): 5.18 (3.53–7.59), *P* < 0.01]. Additionally, within each sub-stage, survival was significantly longer in patients with better liver function (Table 3).

**Table 2:** Factors associated with survival.

Factor	Univariate HR (95% CI)	*P*-value	Multivariate HR (95% CI)	*P-*value
Age (per age)	0.99 (0.99–1.00)	0.74		
Male (vs. female)	1.18 (0.92–1.50)	0.17		
HBV (vs. non-HBV)	1.23 (0.98–1.55)	0.064	1.12 (0.88–1.43)	0.34
Child-Pugh class B (vs. A)	2.05 (1.66–2.52)	<0.001	2.05 (1.64–2.55)	<0.001
Multiple tumor (vs. single)	1.09 (0.92–1.31)	0.29		
Tumor size (≥ 10 vs. < 10cm)	1.75 (1.47–2.09)	<0.001	1.32 (1.08–1.60)	0.005
Portal vein invasion				
None or type I	Reference		Reference	
Type II or type III	1.57 (1.31–1.88)	<0.001	1.63 (1.34–1.99)	<0.001
Extrahepatic spread				
None or lymph node	Reference		Reference	
Distant metastasis	1.73 (1.43–2.11)	<0.001	2.06 (1.65–2.55)	<0.001
AFP (per logAFP)	1.26 (1.18–1.34)	<0.001	1.22 (1.14–1.30)	<0.001

HR, hazard ratio; HBV, hepatitis B virus; AFP, alphafetoprotien.

Multivariate model included etiology (HBV vs. non-HBV), tumor size, portal vein invasion, extrahepatic spread and AFP levels.

**Table 3:** Definition and survival of sub-classified Barcelona Clinic Liver Cancer stage C.

Sub-stage	N	Extent of portal vein invasion	Type of extrahepatic spread	Child-Pugh class	Median survival (months)	*P* value
C1a	198	None or segmental/sectoral	None or nodal	A	13.8	<0.001
C1b	41	None or segmental/sectoral	None or nodal	B	5.8	
C2a	141	Beyond right or left main	None or nodal	A	6.6	0.006
C2b	42	Beyond right or left main	None or nodal	B	3.2	
C3a	98	None or segmental/sectoral	Distant	A	5.2	0.001
C3b	29	None or segmental/sectoral	Distant	B	2.3	
C4a	18	Beyond right or left main	Distant	A	3.6	0.001
C4b	15	Beyond right or left main	Distant	B	1.3	

### Treatment modalities and survival

Treatment modalities that were used in the patients are shown in Table 4. Sorafenib was used as the first line treatment in 7% of patients. Most of the patients (78%) were treated with other modalities, mainly TACE. When compared to patients treated with sorafenib, the median survival was significantly worse for patients managed by best supportive care, and the median survival was significantly better for patients treated with other modalities (Table 4).

**Table 4:** Survival by treatment modalities.

Treatment	No	Median survival (months)	1-year	3-years	*P*-value	Adjusted^*^ Hazard ratio (95% Confidence interval)	*P*-value
**Treatment modalities**					<0.001		
Best supportive care	87 (15%)	2.0	5.8%	0%		1.49 (1.03–2.17)	0.032
Sorafenib	43 (7%)	3.7	6.9%	0%		Reference	
Other modalities	452 (78%)	8.5	41.8%	17.2%		0.39 (0.28–0.55)	<0.001
**Other treatment modalities**					<0.001		
Resection	57	Not reached	69.6%	55.6%		0.12 (0.07–0.20)	<0.001
Transplantation	3	Not reached	66.6%	66.6%		0.07 (0.01–0.52)	0.009
Chemoembolization	345	8.3	41.0%	12.8%		0.39 (0.27–0.54)	<0.001
Systemic chemotherapy	13	3.0	8.3%	0%		1.12 (0.58–2.16)	0.71
Radiation	15	3.7	0%	0%		1.21 (0.66–2.20)	0.53
Experimental	19	5.7	21.0%	0%		0.66 (0.38–1.16)	0.15

^*^Adjusted for age, gender, Child-Pugh class.

Patient survival according to the treatment modalities used in each BCLC sub-stage, with sorafenib as the reference, is shown in Table 5. In sub-stage C1, only three patients received sorafenib treatment as the first-line therapy, hence it could not be used as the reference. Treatment with other modalities in sub-stage C1 showed a relatively good outcome, and the median survival was 14.8 months. In sub-stages C2 and C3, treatment with other modalities resulted in a significantly longer survival than sorafenib. Sorafenib treatment resulted in longer survival compared to the best supportive care, however, the difference did not reach a statistical significance in sub-stages C2 and C3. In sub-stage C4, treatment with sorafenib resulted in longer survival compared to the best supportive care. However, survival was not different between patients treated with sorafenib and patients treated with other modalities. Among 345 patients who received chemoembolization, survival was significantly different according to the sub-stage (median: 12.3, 6.9, 5.6 and 3.6 for C1, C2, C3 and C4, respectively, *P* < 0.001).

**Table 5:** Survival by treatment modalities in each sub-stage.

Treatment	No	Median survival (months)	1-year	*P*-value	Adjusted^*^ HR (95% CI)	*P*-value
**C1 (n = 235)**				<0.001		
Best supportive care	23 (10%)	2.2	9.0%		Reference	-
Sorafenib	3 (0.1%)	4.2	0%		Not tested	
Other modalities^†^	209 (90%)	14.8	54.5%		0.25 (0.15–0.41)	<0.01
TACE	169	12.3	51.0%		0.28 (0.17–0.47)	<0.01
**C2 (n = 178)**				<0.001		
Best supportive care	27 (15%)	2.3	3.7%		1.18 (0.58–2.36)	0.63
Sorafenib	12 (7%)	4.1	0%		Reference	
Other modalities^†^	139 (78%)	6.9	33.8%		0.34 (0.18–0.65)	0.01
TACE	124	6.9	34.6%		0.27 (0.14–0.52)	<0.01
**C3 (n = 118)**				<0.001		
Best supportive care	23 (19%)	2.2	9.1%		1.68 (0.91–3.09)	0.10
Sorafenib	21 (18%)	4.2	14.2%		Reference	
Other modalities^†^	74(63%)	6.3	29.7%		0.54 (0.32–0.91)	0.02
TACE	44	5.6	25.5%		0.52 (0.35–1.08)	0.095
**C4 (n = 32)**				<0.001		
Best supportive care	14 (44%)	1.0	0%		4.23 (1.43–12.45)	0.01
Sorafenib	7 (22%)	3.6	0%		Reference	
Other modalities^†^	11 (34%)	3.6	9.0%		0.89 (0.27–2.91)	0.84
TACE	8	3.6	12.5%		0.37 (0.06–2.15)	0.27

HR, hazard ratio; CI, confidence interval; TACE, transarterial chemoembolization.

^*^Adjusted for age, gender, Child-Pugh class.

^†^Patients who received experimental therapy were excluded.

## Discussion

In this large prospective cohort of patients with BCLC stage C, patient survival varied widely. In some patients, the survival was dismal (median survival of 1.0 month in sub-stage C4 patients who had the best supportive care), while some patients showed a relatively good survival (median survival of 14.8 months in sub-stage C1 patients who were treated with modalities other than sorafenib). The main objective of staging systems is to categorize the patients into subgroups with significantly different outcomes [1]. In this respect, the wide range of survival in BCLC stage C patients indicates the need for sub-classification.

Importantly, this data shows that the extent of PVI should be assessed and staged differently. First, this study observed survival differences based on the extent of PVI. Consistent to this study, previous studies also showed that not just the presence of PVI, but also the extent of PVI is important in determining survival. In the study by Park *et al*., in which 904 patients with HCC were analyzed, the extent of PVI (none, 1^st^ and 2^nd^ branch, and main portal vein) was an independent factor for survival [4]. Shi *et al*. also reported differences in survival based on the extent of macrovascular invasion patterns [7]. The extent of PVI was associated with survival in the report by Mazzaferro et al., as well [16]. Second, in the past, the spatial resolution of CT scans did not allow the detection of most segmental PVI. However, the refinements in imaging techniques that have been introduced in recent years allowed the detection of segmental PVI, and may induce stage migration in patients with segmental portal vein invasion [21]. Indeed, Bolondi *et al*. suggested stage B5 (or quasi-C) to define group of patients with undeterminated peripheral PVI who may be considered as an overlap between intermediate and advanced stages [21]. These findings suggest that minor PVI (limited to sectoral/segmental branch) should not be considered to be the same as macrovascular invasion, indicating that sub-classification of BCLC stage C is needed.

The type of ES was also an independent factor for survival of BCLC stage C patients. In the AJCC/UICC TNM 7^th^ staging system, regional lymph node metastasis (N1) and distant metastasis (M1) is classified separately, while the BCLC staging system does not differentiate between these two types of ES. It is very difficult to assess the effect of ES on survival, because most of these patients die of progressive intrahepatic HCC, and not due to extrahepatic metastasis [22]. Nevertheless, several evidences indicate that nodal and distant metastasis should be classified separately, as patients have different survival according to the type of ES [5]. In the study by Hasegawa *et al*., patients with pathologically proven LN metastasis (any T, N1, M0) had a similar survival to that in patients with advanced T stage (T4, N0, M0), while patients with distant metastasis (any T, any N, M1) had a significantly shorter survival [23].

Therefore, when patients were sub-classified based on these two tumor-related factors (PVI and ES), patients in each sub-stage (C1~C4) showed significantly different outcomes. Significantly different survival based on the extent of PVI and the type of ES indicates that sub-classification of the BCLC stage C will help better predict patient survival.

Staging system should not only assess the prognosis, but it should also help direct therapy. The BCLC staging system is the only staging system used for HCC that has a therapeutic algorithm [3]. The currently recommended treatment for BCLC stage C is sorafenib [3]. However, only 7% of patients were initially treated with sorafenib in this study, due to the late introduction of sorafenib, high costs and strict medical reimbursement policy in Korea, and possibly the physicians’ personal preference. Since this is not a prospective comparative study, selection bias as well as un-identified bias associated with poorer survival in patients treated with sorafenib may exist. Therefore, survival based on treatment modality needs careful interpretation as well as prospective validation. Nevertheless, this study provides some evidences that support the need for sub-classification of BCLC stage C.

First, in sub-stage C1 (patients with segmental/sectoral PVI but without distant metastasis), in which sorafenib was rarely used (0.1%), patients treated with modalities other than sorafenib showed a relatively good survival (median 14.8 months). TACE or radioembolization are not contraindicated in Child-Pugh class A patients with minor PVI [21], and good survival has been reported after TACE [8], and radioembolization with yttrium 90 [16], in this setting. Even further, resection can still be considered, and long-term survival has been reported in patients with minor PVI [24]. Therefore, in patients with segmental/sectoral PVI, relatively long-term survival can be expected after treatment other modalities (radioembolization, chemoembolization, resection etc.), and sorafenib may not be a first-line option.

Secondly, a wide range of treatment modalities other than sorafenib are being used for patients with PVI in daily practice in the real-world setting [25]. Furthermore, some selected patients with ES receive a combination of locoregional therapies for both the intrahepatic HCC and symptomatic ES, or a combination of locoregional therapies with sorafenib, and they have a relatively good survival [22, 26]. The present study, based on survival analysis, demonstrated the advantage of other treatment modalities over sorafenib in sub-stages C2 and C3. However, and advantage of other treatment modalities was not observed in sub-stage C4 compared to sorafenib treatment. Although, prospective comparative studies including head to head comparison of sorafenib with other modalities (resection, radioembolization or TACE and/or sorafenib) are needed to validate this finding, this suggests that careful selection of patients for a multidisciplinary treatment approach may result in a change in the prognosis in some BCLC stage C patients toward a relatively favorable long-term survival. There is also a concern that due to the lack of prospective comparative studies, both heterogeneity and role of each therapeutic modality in advanced BCLC stage are incompletely captured and translated into recommendations in the BCLC guidelines [24]. Therefore, in real clinical practice, different treatment algorithms for BCLC stage C are being used in many tertiary centers [27]. By sub-classifying advanced HCC patients, comparison between studies can be easier, and may help to guide the clinicians in selecting the most appropriate therapy.

There are some limitations in this study. In this study, when patients were further classified based on Child-Pugh class (A or B), better prognostic information was acquired. We observed a survival difference by further sub-classifying patients according to the liver function (e.g., C1a or C1b). However, due to the relatively small number of patients in each arm, we could not analyze whether this approach will help in deciding further treatment plans. Patients in this study are mostly hepatitis B virus-related HCC, and all Asian. Nodal and distant metastasis was mostly defined radiologically, and not all cases were pathologically proven cases.

In summary, BCLC stage C includes a heterogeneous patient population in terms of tumor burden and liver function, and poses a unique challenge for therapeutic management. Patient survival is significantly different according to the extent of PVI and the type of ES in the same stage. Although there is proven benefit from sorafenib treatment in this setting [17], various other treatments are used in the real world on an empirical basis, and show a better outcome than sorafenib in selected patients. Therefore, our data suggest that BCLC stage C needs to be sub-classified to better predict the prognosis and to guide most appropriate treatment.

## References

[1] Forner A, Llovet JM, Bruix J. Hepatocellular carcinoma. Lancet. 2012;379: 1245–1255. 10.1016/S0140-6736(11)61347-0 22353262 PMC13537732

[2] Osaki Y, Nishikawa H. Treatment for hepatocellular carcinoma in Japan over the last three decades: Our experience and published work review. Hepatol Res. 2015;45: 59–74. 10.1111/hepr.12378 24965914 PMC4313689

[3] Bruix J, Sherman M. Management of hepatocellular carcinoma: an update. Hepatology. 2011;53: 1020–1022. 10.1002/hep.24199 21374666 PMC3084991

[4] Park KW, Park JW, Choi JI, Kim TH, Kim SH, Park HS, et al Survival analysis of 904 patients with hepatocellular carcinoma in a hepatitis B virus-endemic area. J Gastroenterol Hepatol. 2008;23: 467–473. 17764529 10.1111/j.1440-1746.2007.05112.x

[5] Kee KM, Wang JH, Lin CY, Wang CC, Cheng YF, Lu SN. Validation of the 7th edition TNM staging system for hepatocellular carcinoma: an analysis of 8,828 patients in a single medical center. Dig Dis Sci. 2013;58: 2721–2728. 10.1007/s10620-013-2716-8 23703450

[6] Yang T, Lin C, Zhai J, Shi S, Zhu M, Zhu N, et al Surgical resection for advanced hepatocellular carcinoma according to Barcelona Clinic Liver Cancer (BCLC) staging. J Cancer Res Clin Oncol. 2012;138: 1121–1129. 10.1007/s00432-012-1188-0 22402598 PMC11824283

[7] Shi J, Lai EC, Li N, Guo WX, Xue J, Lau WY, et al Surgical treatment of hepatocellular carcinoma with portal vein tumor thrombus. Ann Surg Oncol. 2010;17: 2073–2080. 10.1245/s10434-010-0940-4 20131013

[8] Prajapati HJ, Dhanasekaran R, El-Rayes BF, Kauh JS, Maithel SK, Chen Z, et al Safety and efficacy of doxorubicin drug-eluting bead transarterial chemoembolization in patients with advanced hepatocellular carcinoma. J Vasc Interv Radiol. 2013;24: 307–315. 10.1016/j.jvir.2012.11.026 23375519

[9] Park JY, Ahn SH, Yoon YJ, Kim JK, Lee HW, Lee do Y, et al Repetitive short-course hepatic arterial infusion chemotherapy with high-dose 5-fluorouracil and cisplatin in patients with advanced hepatocellular carcinoma. Cancer. 2007;110: 129–137. 17508408 10.1002/cncr.22759

[10] Memon K, Kulik L, Lewandowski RJ, Mulcahy MF, Benson AB, Ganger D, et al Radioembolization for hepatocellular carcinoma with portal vein thrombosis: impact of liver function on systemic treatment options at disease progression. J Hepatol. 2013;58: 73–80. 10.1016/j.jhep.2012.09.003 23000237 PMC3527660

[11] Yoon EL, Yeon JE, Lee HJ, Suh SJ, Lee SJ, Kang SH, et al Systemic cytotoxic chemotherapy of patients with advanced hepatocellular carcinoma in the era of sorafenib nonavailability. J Clin Gastroenterol. 2014;48: e22–29. 10.1097/MCG.0b013e3182a54ec8 24045282

[12] Cho JY, Paik YH, Park HC, Yu JI, Sohn W, Gwak GY, et al The feasibility of combined transcatheter arterial chemoembolization and radiotherapy for advanced hepatocellular carcinoma. Liver Int. 2014;34: 795–801. 10.1111/liv.12445 24350564

[13] Choi GH, Shim JH, Kim MJ, Ryu MH, Ryoo BY, Kang YK, et al Sorafenib alone versus sorafenib combined with transarterial chemoembolization for advanced-stage hepatocellular carcinoma: results of propensity score analyses. Radiology. 2013;269: 603–611. 10.1148/radiol.13130150 23864102

[14] Cha J, Seong J, Lee IJ, Kim JW, Han KH. Feasibility of sorafenib combined with local radiotherapy in advanced hepatocellular carcinoma. Yonsei Med J. 2013;54: 1178–1185. 10.3349/ymj.2013.54.5.1178 23918567 PMC3743177

[15] Yoo EJ, Shin HS, Kim SU, Joo DJ, Park JY, Choi GH, et al Orthotopic liver transplantation after the combined use of locoregional therapy and sorafenib for advanced hepatocellular carcinoma. Onco Targets Ther. 2013;6: 755–759. 10.2147/OTT.S45602 23836988 PMC3699313

[16] Mazzaferro V, Sposito C, Bhoori S, Romito R, Chiesa C, Morosi C, et al Yttrium-90 radioembolization for intermediate-advanced hepatocellular carcinoma: a phase 2 study. Hepatology. 2013;57: 1826–1837. 10.1002/hep.26014 22911442

[17] Llovet JM, Ricci S, Mazzaferro V, Hilgard P, Gane E, Blanc JF, et al Sorafenib in advanced hepatocellular carcinoma. N Engl J Med. 2008;359: 378–390. 10.1056/NEJMoa0708857 18650514

[18] Cheng AL, Kang YK, Chen Z, Tsao CJ, Qin S, Kim JS, et al Efficacy and safety of sorafenib in patients in the Asia-Pacific region with advanced hepatocellular carcinoma: a phase III randomised, double-blind, placebo-controlled trial. Lancet Oncol. 2009;10: 25–34. 10.1016/S1470-2045(08)70285-7 19095497

[19] Korean Liver Cancer Study Group and National Cancer Center. Practice guidelines for management of hepatocellular carcinoma 2009. Korean J Hepatol. 2009;15: 391–423. 10.3350/kjhep.2009.15.3.391 19783891

[20] Lencioni R, Llovet JM. Modified RECIST (mRECIST) assessment for hepatocellular carcinoma. Semin Liver Dis. 2010;30: 52–60. 10.1055/s-0030-1247132 20175033 PMC12268942

[21] Bolondi L, Burroughs A, Dufour JF, Galle PR, Mazzaferro V, Piscaglia F, et al Heterogeneity of patients with intermediate (BCLC B) Hepatocellular Carcinoma: proposal for a subclassification to facilitate treatment decisions. Semin Liver Dis. 2012;32: 348–359. 10.1055/s-0032-1329906 23397536

[22] Lee HS. Management of patients with hepatocellular carcinoma and extrahepatic metastasis. Dig Dis. 2011;29: 333–338. 10.1159/000327572 21829026

[23] Hasegawa K, Makuuchi M, Kokudo N, Izumi N, Ichida T, Kudo M, et al Impact of histologically confirmed lymph node metastases on patient survival after surgical resection for hepatocellular carcinoma: report of a Japanese nationwide survey. Ann Surg. 2014;259: 166–170. 10.1097/SLA.0b013e31828d4960 23532111

[24] Cillo U. Liver resection is a therapeutic option for highly selected BCLC C patients in the context of an expert multidisciplinary setting. Dig Liver Dis. 2013;45: 460–461. 10.1016/j.dld.2013.03.010 23660077

[25] Kim do Y, Han KH. How to improve treatment outcomes for hepatocellular carcinoma of intermediate and advanced stage. Dig Dis. 2012;30: 598–602. 10.1159/000343088 23258101

[26] Lee S, Kim BK, Kim SU, Park Y, Chang S, Park JY, et al Efficacy of sorafenib monotherapy versus sorafenib-based loco-regional treatments in advanced hepatocellular carcinoma. PLoS One. 2013;8: e77240 10.1371/journal.pone.0077240 24155932 PMC3796498

[27] Livraghi T, Brambilla G, Carnaghi C, Tommasini MA, Torzilli G. Is it time to reconsider the BCLC/AASLD therapeutic flow-chart? J Surg Oncol. 2010;102: 868–876. 10.1002/jso.21733 20886553

